# Levels of acylation stimulating protein and the complement component 3 precursor are associated with the occurrence and development of coronary heart disease

**DOI:** 10.3892/etm.2014.2018

**Published:** 2014-10-13

**Authors:** HONGLEI JIANG, MIAO GUO, LINPING DONG, CHUNLIN CAO, DONG WANG, XIAOTANG LIANG, FANG GUO, ZHAOQIN XING, PEILI BU, JIDONG LIU

**Affiliations:** 1 Department of Cardiology, Provincial Hospital of Shandong University, Jinan, Shandong 250021, P.R. China; 2 Department of Gerontology, Linyi People’s Hospital, Linyi, Shandong 276000, P.R. China; 3 Department of Orthopedics, Provincial Hospital of Shandong University, Jinan, Shandong 250021, P.R. China; 4 Key Laboratory of Cardiovascular Remodeling and Function Research of Chinese Ministry of Education and Chinese Ministry of Health, Department of Cardiology, Qilu Hospital of Shandong University, Jinan, Shandong 250012, P.R. China

**Keywords:** metabolic syndrome, coronary heart disease, atherosclerosis, acylation stimulating protein, complement component 3

## Abstract

The aim of the present study was to investigate whether acylation stimulating protein (ASP) and complement component 3 (C3) are associated with the occurrence and development of coronary heart disease (CHD). The participants of the study were divided into three groups, including the healthy control (n=42), metabolic syndrome (MS, n=56) and CHD (n=62) groups. An enzyme-linked immunosorbent assay was used to measure the ASP concentrations, while an immunoturbidimetric assay was employed to determine the C3 concentrations. In addition, coronary angiography was performed to determine the severity of coronary artery disease in the CHD group. The CHD group was divided into three subgroups, according to the final Gensini score of coronary artery stenosis for each patient (mild, ≤20 points; moderate, 21–40 points; severe, >40 points). Western blotting and quantitative reverse transcription-polymerase chain reaction (RT-PCR) were performed to analyze the protein and mRNA expression levels of C3 in the CHD subgroups and the healthy control group. The concentrations of ASP and C3 in the CHD group were found to be significantly higher compared with the control and MS groups. In addition, the levels of ASP and C3 in the mild and moderate CHD subgroups were significantly higher compared with the healthy controls or mild CHD patients. Furthermore, the protein expression levels of C3 in the moderate and severe CHD patients were found to be significantly higher compared with the healthy individuals and the mild CHD patients. The quantitative RT-PCR results revealed that the mRNA expression levels of C3 in the moderate and severe CHD patients were significantly higher compared with the healthy control group and the mild CHD patients. Furthermore, the mean levels of C3 transcripts in the severe CHD patients were found to be higher compared with the moderate CHD subgroup (P<0.05). Therefore, ASP and C3 were found to be associated with the occurrence and development of CHD; thus, may be used as novel indexes for CHD.

## Introduction

Recent studies have indicated that atherosclerosis is closely associated with adipokines (1,2). Acylation stimulating protein (ASP) is a source of adipokine derived from complement component 3 (C3) (3). As a core factor of the complement system, C3 functions as a key molecule, initiating an alternative pathway for complement activation and participating in cascades. In addition, C3 is considered to be a link between atherosclerosis and high blood pressure, high cholesterol, insulin resistance or obesity, functioning as an independent index for atherosclerosis risk factors (4–6). Endogenous C3 is mainly derived from fat tissues and the liver (7). In a physiological state, chylomicrons stimulate the secretion of C3 from adipose cells. ASP is a cleaved fragment of C3 that functions as a strong stimulating factor, promoting fatty acid esterification and triglyceride synthesis. In addition, ASP is a bioactive substance, closely associated with human fat metabolism (8).

ASP metabolic pathway dysfunction causes a number of carbohydrate and lipid metabolic disorders, leading to obesity, diabetes and cardiovascular diseases (9). However, studies investigating the correlation of ASP and C3 with atherosclerosis have been rarely reported.

In the present study, the role of ASP in the occurrence and development of atherosclerosis was investigated by analyzing the levels of ASP in healthy controls and patients with coronary heart disease (CHD) or metabolic syndrome (MS). In addition, the brachial-ankle pulse wave velocity (baPWV) and ankle-brachial index (ABI) of patients suffering from MS were determined, and the correlation with the ASP and C3 concentration was determined. Furthermore, the correlation between the ASP and C3 concentration with the severity of coronary artery disease in CHD patients was analyzed to determine whether ASP is a risk factor in the occurrence and development of CHD. The CHD patients were divided into mild, moderate and severe CHD groups, according to coronary artery stenosis grading. The protein and mRNA expression levels of C3 in the mild, moderate and severe CHD subgroups were determined by western blotting and quantitative reverse transcription-polymerase chain reaction (RT-PCR), respectively.

## Materials and methods

### Subjects

A total of 118 patients diagnosed with CHD in the Department of Cardiology of Provincial Hospital of Shandong (Jinan, China) were enrolled in the study and divided into the MS (n=56) and CHD (n=62) groups. MS patients (male, 27; female, 29) were diagnosed according to the MS diagnostic criteria (10). Patients suffering from heart failure, acute coronary syndrome, an infectious disease, rheumatism, cancer and secondary MS, or patients administered anticoagulants, were excluded from the MS group. In the CHD patients (male, 30; female, 32), the degree of vascular stenosis was determined by coronary angiography. Patients suffering from severe infections or connective tissue disease were excluded from the CHD group. In addition, 42 healthy volunteers (male, 20; female, 22) were recruited for the control group. The individuals in the three groups were aged between 45 and 81 years. No statistically significant differences existed in the age and gender compositions between the groups. The study was approved by the Ethics Committee of the Provincial Hospital of Shandong University (Jinan, China) and written informed consent was obtained from the patient.

### Arteriosclerosis detection

An arteriosclerosis detection device BP-203RPEIII (Omron Healthcare Co., Ltd, Tokyo, Japan) was used to determine the baPWV and ABI values of the patients, measured after resting in the supine position for >5 min. Basic information of the individuals, including height, weight and age, were inserted in the device. In addition, heart sounds were recorded from the fourth intercostal space of the left sternal border, while electrocardiogram electrodes were fixed around the wrists, and four cuffs, connected with a plethysmograph and oscillometric pressure sensor, were placed on the ankle brachial artery. Following stabilization of the phonocardiogram signal, the average baPWV and ABI values were determined by recording three consecutive measurements. The highest bilateral limb value of baPWV and the lowest bilateral limb value of ABI were selected for analysis. baPWV values of <1,400 cm/sec were considered as normal reference values, while values of ≥1,400 cm/sec were regarded as abnormal. With regard to ABI, values between 0.9 and 1.4 were considered to be normal, values of ≤0.9 indicated that patients may suffer from arteriosclerotic occlusion in the lower limbs and values of ≥1.4 indicated the possibility of blood vessel calcification.

### Coronary angiography

Selective coronary angiography was performed using Judkins method. Quantitative analysis of the vascular stenosis degree was performed according to the Gensini scoring system (11), as follows: 1 point, ≤25% stenosis; 2 points, 26–50% stenosis; 4 points, 51–75% stenosis; 8 points, 76–90% stenosis; 16 points, 91–99% stenosis; and 32 points, 100% stenosis. Different segments of the coronary artery were multiplied with appropriate factors. The final score of coronary artery stenosis for each patient was the sum of all the branch points. According to the final scores, the individuals were divided into the four groups as follows: Healthy control (0 points), mild CHD (≤20 points), moderate CHD (21–40 points) and severe CHD (>40 points) groups.

### Enzyme-linked immunosorbent assay (ELISA) and immunoturbidimetry

Fasting venous blood samples were collected from the subjects, and the serum was separated by immediate centrifugation at 1,411 × g at 4°C for 10 min. Serum aliquots were stored at −20°C for further use. The serum ASP concentration was measured using an OmniKine™ Human ACE ELISA kit (Assay Biotech Co., Inc., Sunnyvale, CA, USA), according to the manufacturer’s instructions. Immunoturbidimetry was performed to measure the concentration of C3 using an Immunoturbidimetry kit (Assay Biotech Co., Inc.).

### Immunoblot assays

Total proteins were isolated from the serum of each participant, separated by 10% sodium dodecyl sulfate/polyacrylamide gel electrophoresis and subjected to immunoblot analyses. Primary antibodies against C3 and actin were purchased from Santa Cruz Biotechnology, Inc. (Dallas, TX, USA; anti-C3, cat no. sc-28294, 1:200; anti-actin, cat no. sc-130301, 1:10,000). A secondary goat anti-mouse immunoglobulin G-horseradish peroxidase-conjugated antibody was also used (Santa Cruz Biotechnology, Inc.; cat no. sc-2005, 1:10,000). The signals were detected using an enhanced chemiluminescence system (Pierce Biotechnology, Inc., Rockford, IL, USA), and immunoblotting experiments were performed a minimum of three times. The mean optical density (OD) of the C3 protein bands was normalized against the OD of the actin band of each individual.

### Quantitative RT-PCR

Quantitative RT-PCR analysis was performed to determine the mRNA expression levels of C3 and GAPDH. The total RNA was harvested from the serum of all individuals using an RNeasy kit (Qiagen, Valencia, CA, USA), according to the manufacturer’s instructions. Briefly, the PCR procedures were as follows: Pre-denaturation at 95°C for 30 sec, followed by 35 cycles of denaturation at 95°C for 15 sec and annealing at 56°-60°C for 30 sec. RT-PCR experiments were performed a minimum of three times.

RNA (1 μl) was reverse transcribed into cDNA using random primers in a Reverse Transcription II system (Promega Corporation, Madison, WI, USA), according to the manufacturer’s instructions. The mRNA expression levels of C3 and GAPDH were determined using quantitative PCR with an ABI Prism Sequence Detection system (Applied Biosystems Life Technologies, Foster City, CA, USA). The primers used are listed in Table I. An assay reagent containing premixed primers and a VIC-labeled probe (Applied Biosystems Life Technologies; cat no. 4310884E) was used to quantify the mRNA expression level of endogenous GAPDH. The relative levels of C3 transcripts were normalized against the amount of GAPDH mRNA for each sample.

### Statistical analysis

Statistical analyses were performed using the Statistical Package for Social Sciences software (SPSS version 13.0; SPSS, Inc., Chicago, IL, USA). The data are expressed as the mean ± standard deviation. Comparisons between two groups were performed using the t-test, where P<0.05 was considered to indicate a statistically significant difference.

## Results

### ASP and C3 concentrations are significantly increased in CHD patients compared with healthy individuals and MS patients

**Figure 1:**
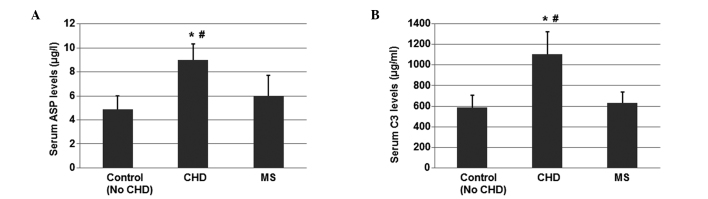
Levels of (A) ASP and (B) C3, determined by ELISA and immunoturbidimetry, respectively, using fasting venous blood samples collected from patients in the control, CHD and MS groups. ^*^P<0.05, vs. healthy control group; ^#^P<0.05, vs. MS group. ASP, acylation stimulating protein; C3, complement component 3; CHD, coronary heart disease; MS, metabolic syndrome; ELISA, enzyme-linked immunosorbent assay.

ELISA was used to determine the serum concentrations of ASP in the control, CHD and MS groups. As shown in Fig. 1A, the levels of ASP in the CHD group were significantly increased compared with the MS and control groups (P<0.05). However, the ASP levels in the MS group were not found to be significantly different when compared with the control group (P>0.05). These results indicate that the concentration of ASP in CHD patients was significantly enhanced compared with the control and MS groups.

Immunoturbidimetric analysis was performed to determine the serum concentrations of C3 in the control, CHD and MS groups (Fig. 1B). As shown in Fig. 1B, the levels of C3 in the CHD group were significantly increased compared with the MS and control groups (P<0.05). However, the difference in the C3 levels between the MS and control groups was not found to be statistically significant (P>0.05). These results indicate that the concentration of C3 in CHD patients was significantly increased compared with the control and MS patients. Thus, ASP and C3 levels may correlate with the occurrence of CHD, but not MS.

### ASP and C3 levels are slightly increased in MS patients with abnormal baPWV and ABI readings

An arteriosclerosis detection device was used to determine whether the levels of ASP and C3 were altered in MS patients with abnormal baPWV and ABI readings. As shown in Table II, the levels of ASP and C3 in the MS patients with abnormal baPWV and ABI readings were slightly increased compared with MS patients with normal baPWV and ABI values. There were no statistically significant differences in the levels of ASP (P=0.306) and C3 (P=0.416) between patients with normal and abnormal baPWV values. Additionally, the differences in the levels of ASP (P=0.406) and C3 (P=0.504) between patients with normal and abnormal ABI values were not significant. The results indicate that the ASP and C3 levels were not significantly altered in MS patients with abnormal baPWV and ABI readings.

### ASP and C3 levels are positively correlated with the severity of coronary artery disease in the CHD group

Coronary angiography and Gensini scoring were used to further evaluate the vascular stenosis degree of CHD patients, and determine the correlation between ASP levels and the degree of coronary artery disease. There were 10 cases with a Gensini score of 0 points and 12 cases with a Gensini score ≤ 20 points. The Gensini score of 22 cases was between 20 and 40 points. Additionally, a total of 18 cases had a Gensini score ≥ 40 points.

**Figure 2:**
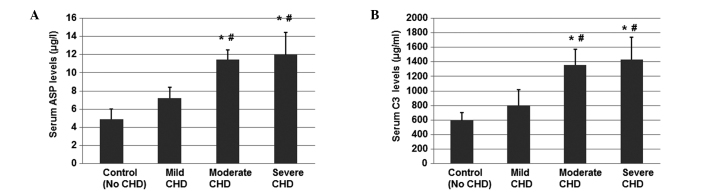
Levels of (A) ASP and (B) C3, determined by ELISA and immunoturbidimetry, respectively, using fasting venous blood samples collected from patients in the control group (n=42) and mild, moderate and severe CHD subgroups (n=62). ^*^P<0.05, vs. healthy control group; ^#^P<0.05, vs. mild CHD subgroup. ASP, acylation stimulating protein; C3, complement component 3; CHD, coronary heart disease; ELISA, enzyme-linked immunosorbent assay.

ELISA was performed to determine the serum concentrations of ASP in the three CHD subgroups (mild, moderate and severe) and the healthy control group. As shown in Fig. 2A, the levels of ASP in the moderate and severe CHD subgroups were significantly higher compared with the control group and mild CHD subgroup (P<0.05). However, the ASP levels were not found to be significantly different between the moderate and severe CHD subgroups. These results indicate that the levels of ASP in CHD patients correlate with the severity degree of CHD.

Since C3 is the precursor of ASP, the levels of C3 in the CHD patients were also measured using immunoturbidimetry. As shown in Fig. 2B, the levels of C3 in the patients with moderate and severe CHD were significantly higher compared with the control group and the mild CHD subgroup (P<0.05). In addition, the C3 levels in the moderate and severe CHD subgroups were similar (P>0.05). These results indicate that the levels of C3 in CHD patients correlate with the severity of CHD.

### Protein expression levels of C3 in CHD patients are significantly increased compared with healthy individuals

**Figure 3:**
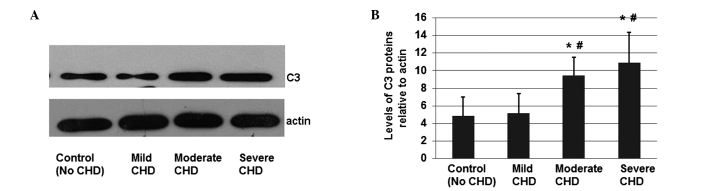
(A) Representative immunoblot and (B) histogram showing the mean OD of C3 protein bands normalized against the OD of the actin band of each participant. Error bars show the standard deviation of the mean (mean ± standard deviation). ^*^P<0.05, vs. healthy control group; ^#^P<0.05, vs. mild CHD subgroup. OD, optical density; C3, complement component 3; CHD, coronary heart disease.

Total proteins were extracted from individuals in the healthy control group and mild, moderate and severe CHD subgroups, in order to investigate whether the protein expression of C3 was altered in CHD patients. The protein expression levels of C3 and actin were determined by western blotting (Fig. 3A), and the mean OD of the C3 protein bands was normalized against the OD of the actin band of each participant. Error bars in Fig. 3B show the standard error of the mean (mean ± SD; P<0.05).

As shown in Fig. 3, the mean protein expression levels of C3 in the moderate and severe CHD subgroups were significantly higher compared with healthy individuals and the mild CHD subgroup, indicating that protein expression of C3 is associated with the development of CHD.

### mRNA expression levels of C3 in CHD patients are significantly increased compared with healthy individuals

High protein expression levels are often a result of a high level of gene transcription. Therefore, the serum mRNA expression levels of C3 in the healthy control group and the three CHD subgroups were determined using quantitative RT-PCR analysis. The fold change was calculated by normalizing the level of C3 transcripts to those in the control group. The mean level of the C3 transcripts in the healthy control group was assigned a value of 1,000.

**Figure 4:**
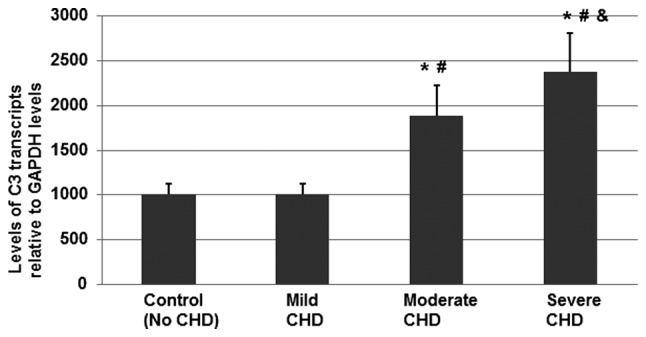
Quantitative reverse transcription-polymerase chain reaction analysis of the mRNA expression level of C3 in the healthy control group (n=42) and the three CHD subgroups (n=62). The C3 transcript levels were normalized against GADPH. ^*^P<0.05, vs. healthy control group; ^#^P<0.05, vs. mild CHD subgroup; ^&^P<0.05, vs. moderate CHD subgroup. C3, complement component 3; CHD, coronary heart disease.

As shown in Fig. 4, the mean expression levels of C3 mRNA in the moderate and severe CHD patients were significantly higher compared with the healthy control group and the mild CHD subgroup (P<0.05). In addition, the mean mRNA expression levels of C3 in the severe CHD patients were found to be significantly higher compared with the moderate CHD subgroup (P<0.05). Therefore, C3 gene transcription was found to be associated with the development of mild CHD to moderate CHD.

## Discussion

Early diagnosis and treatment of cardiovascular diseases is important for the improvement of prognosis and the reduction of morbidity and mortality rates. Decreased arterial elasticity is an important link that connects the underlying disease, risk factors and disease endpoints in the cardiovascular event chain. Timely identification and knowledge of the degree of arteriosclerosis, and subsequent active intervention, have become a priority in prevention of morbidity and mortality (12).

baPWV and ABI are frequently used in the evaluation of early atherosclerosis. A number of studies have confirmed that a baPWV value of >1,800 cm/sec indicates the occurrence of severe coronary events (13,14); therefore, baPWV can be used to diagnose CHD. In addition, ABI and baPWV contribute towards the early detection of changes in the vascular structure and function. However, the present study revealed that ASP and C3 were not correlated with baPWV or ABI. Carotid atherosclerosis is an indicator of systemic atherosclerosis, and is closely associated with the incidence of cardiovascular and cerebrovascular diseases (15). Ultrasound has become the preferred imaging examination method for patients with carotid atherosclerosis, with numerous studies confirming that the presence of carotid atherosclerosis is a valuable independent predictor of coronary atherosclerosis (16–18). In the current study, the protein expression levels of C3 in the moderate and severe CHD patients were found to be significantly higher compared with the healthy individuals and mild CHD patients. In addition, quantitative RT-PCR analysis indicated that the levels of C3 mRNA in the moderate and severe CHD patients were significantly higher compared with the healthy individuals and the mild CHD patients. Furthermore, the mean transcript levels of C3 in the severe CHD patients were found to be higher compared with the moderate CHD subgroup (P<0.05). In the present study, an association between ASP and C3 with moderate and severe CHD was observed, indicating that the factors may be involved in the occurrence and development of atherosclerosis.

ASP receptor dysfunction is known to exist in patients with CHD, contributing to the decline of the effect of ASP (19). In addition, ASP dysfunction results in reduced triglyceride synthesis in adipose cells, with the majority of free fatty acids and cholesterol particles, consisting of triglycerides, being retained in the circulation for subsequent uptake by the liver (20). In order to transport the increased fatty acid molecules, an increased number of very-low-density lipoprotein particles, containing the abundant apolipoprotein B100, are synthesized and released by the liver, further aggravating the disorder of lipid metabolism (21).

In conclusion, the present study revealed a significant correlation between the levels of ASP and C3 with the severity of coronary arteriosclerosis, indicating that ASP is involved in the progress of CHD. An in-depth study of the biological function and metabolic pathways of ASP may aid further elucidation of the mechanism underlying the occurrence of CHD with respect to lipid metabolism disorders, and provide a potential method for the prevention and treatment of cardiovascular diseases.

## Figures and Tables

**Table I:** Primers used in the study.

Primers	Primer sequences
C3_F	5′-AGAATCGAACATAGACAGATAGT-3′
C3_R	5′-CTTCGTCATGGTCATGTAGAAC-3′
GAPDH_F	5′-TCGCTCTTACAAGTCGATCCA-3′
GAPDH_R	5′-GACCAAGCTGACTCGTAGCT-3′

C3, complement component 3; F, forward; R, reverse.

**Table II:** Levels of ASP and C3 in the MS group.

Subgroup	ASP (μg/l)	P-value	C3 (μg/ml)	P-value
baPWV values		0.306		0.416
Normal (n=30)	4.78±1.33		619.4±185.7	
Abnormal (n=32)	4.86±1.68		639.7±165.5	
ABI values		0.406		0.504
Normal (n=28)	4.99±1.13		631.4±151.6	
Abnormal (n=34)	5.01±1.23		638.5±145.6	

ASP, acylation-stimulating protein; C3, complement component 3; MS, metabolic syndrome; baPWV, brachial-ankle pulse wave velocity; ABI, ankle-brachial index. The diferences in the levels of ASP and C3 between patients with normal and abnormal baPWV values and between patients with normal and abnormal ABI values were not statistically significant (P>0.05).

## References

[1] Elks CM, Francis J (2010). Central adiposity, systemic inflammation, and the metabolic syndrome. Curr Hypertens Rep.

[2] Gremese E, Ferraccioli G (2011). The metabolic syndrome: the crossroads between rheumatoid arthritis and cardiovascular risk. Autoimmun Rev.

[3] Cianflone KM, Sniderman AD, Walsh MJ (1989). Purification and characterization of acylation stimulating protein. J Biol Chem.

[4] Capuano V, D’Arminio T, La Sala G, Mazzotta G (2006). The third component of the complement (C3) is a marker of the risk of atherogenesis. Eur J Cardiovasc Prev Rehabil.

[5] Engström G, Hedblad B, Eriksson KF (2005). Complement C3 is a risk factor for the development of diabetes: a population-based cohort study. Diabetes.

[6] Weyer C, Tataranni PA, Pratley RE (2000). Insulin action and insulinemia are closely related to the fasting complement C3, but not acylation stimulating protein concentration. Diabetes Care.

[7] Gabrielsson BG, Johansson JM, Lönn M (2003). High expression of complement components in omental adipose tissue in obese men. Obes Res.

[8] MacLaren R, Cui W, Cianflone K (2008). Adipokines and the immune system: an adipocentric view. Adv Exp Med Biol.

[9] Maslowska M, Wang HW, Cianflone K (2005). Novel roles for acylation stimulating protein/C3adesArg: a review of recent in vitro and in vivo evidence. Vitam Horm.

[10] Metabolic syndrome study cooperation group of Chinese diabetes society (2004). Suggestions about metabolic syndrome of Chinese diabetes society. Zhong Hua Tang Niao Bing Za Zhi.

[11] Gensini GG (1983). A more meaningful scoring system for determining the severity of coronary heart disease. Am J Cardiol.

[12] Rubba F, Gentile M, Iannuzzi A (2010). Vascular preventive measures: the progression from asymptomatic to symptomatic atherosclerosis management. Evidence on usefulness of early diagnosis in women and children. Future Cardiol.

[13] Yamashina A, Tomiyama H, Arai T (2003). Brachial-ankle pulse wave velocity as a marker of atherosclerotic vascular damage and cardiovascular risk. Hypertens Res.

[14] Xu Y, Wu Y, Li J, Ma W, Guo X, Luo Y, Hu D (2008). The predictive value of brachial-ankle pulse wave velocity in coronary atherosclerosis and peripheral artery diseases in urban Chinese patients. Hypertens Res.

[15] Faxon DP, Creager MA, Smith SC, American Heart Association (2004). Atherosclerotic vascular disease conference: Executive summary: Atherosclerotic vascular disease conference proceeding for healthcare professionals from a special writing group of the American Heart Association. Circulation.

[16] Hulthe J, Wikstrand J, Emanuelsson H (1997). Atherosclerotic changes in the carotid artery bulb as measured by B-mode ultrasound are associated with the extent of coronary atherosclerosis. Stroke.

[17] Yildiz A, Tepe S, Oflaz H (2004). Carotid atherosclerosis is a predictor of coronary calcification in chronic haemodialysis patients. Nephrol Dial Transplant.

[18] Kurnatowska I, Grzelak P, Stefańczyk L, Nowicki M (2010). Tight relations between coronary calcification and atherosclerotic lesions in the carotid artery in chronic dialysis patients. Nephrology (Carlton).

[19] Sniderman AD, Cianflone K, Arner P, Summers LK, Frayn KN (1998). The adipocyte, fatty acid trapping, and atherogenesis. Arterioscler Thromb Vasc Biol.

[20] Hajer GR, van Haeften TW, Visseren FL (2008). Adipose tissue dysfunction in obesity, diabetes, and vascular diseases. Eur Heart J.

[21] Adiels M, Olofsson SO, Taskinen MR, Borén J (2008). Overproduction of very low-density lipoproteins is the hallmark of the dyslipidemia in the metabolic syndrome. Arterioscler Thromb Vasc Biol.

